# ECHO: Results from a Non-Interventional Cohort Study of Esketamine Nasal Spray in Treatment Resistant Depression

**DOI:** 10.1192/j.eurpsy.2026.10606

**Published:** 2026-07-31

**Authors:** G. Rosso, E. Constant, S. De Filippis, Y. Godinov, L. Häggström, A. Ibañez, Y. Kambarov, E. Olivier, J. M. Olivares, C. Reif-Leonhard, B. Rive, I. Turkoz, C. von Holt, D. Zeeuws, S. Köhler

**Affiliations:** 1 Department of Neurosciences, San Luigi Gonzaga Hospital, University of Turin, Turin, Italy; 2 Centre Hospitalier Spécialisé Notre-Dame des Anges, Liège; 3 Université Catholique de Louvain, Brussels; 4 Université de Liège, Liège, Belgium; 5 Neuropsychiatric Clinic Villa Von Siebenthal, Genzano di Roma, Rome, Italy; 6 Johnson & Johnson, Sofia, Bulgaria; 7 Affecta Psychiatric Clinic, Halmstad, Sweden; 8 Department of Psychiatry, Hospital Universitario Ramón y Cajal, Universidad de Alcala, Instituto Ramón y Cajal de Investigación Sanitaria (IRYCIS), Ciber de Salud Mental (CIBERSAM), ISCII, Madrid, Spain; 9 Johnson & Johnson, Beerse, Belgium; 10 APareeGGZ, Rotterdam, Netherlands; 11 Health Research Institute Galicia Sur (IISGS, CIBERSAM); 12 Psychiatry Service of the Health Area of Vigo, Vigo, Spain; 13 Department of Psychiatry, Psychosomatic Medicine and Psychotherapy, University Hospital Frankfurt – Goethe University Frankfurt, Frankfurt am Main, Germany; 14 Johnson & Johnson, Paris, France; 15 Johnson & Johnson, Titusville, New Jersey, United States; 16 Johnson & Johnson, Neuss, Germany; 17 Department of Psychiatry, Universitair Ziekenhuis Brussel; 18 Center for Neurosciences (C4N), Vrije, Universiteit Brussel (VUB), Brussels, Belgium; 19 Alexianer St. Joseph Hospital Berlin-Weißensee; 20 Charité Universitätsmedizin, Berlin, Germany

## Abstract

**Introduction:**

Esketamine nasal spray (ESK-NS) in combination with a selective serotonin reuptake inhibitor/serotonin-norepinephrine reuptake inhibitor is indicated for treatment of adults with treatment resistant depression (TRD) lacking response to ≥2 pharmacological treatments in the current moderate to severe depressive episode (Spravato SmPC; EMA). The ECHO study investigated ESK-NS efficacy and safety in real-world clinical settings.

**Objectives:**

To present the first results of the ECHO study, which included patients (pts) with TRD initiating ESK-NS treatment in 7 participating countries.

**Methods:**

ECHO was a prospective, international, non-interventional phase IV study. Eligible pts were adults diagnosed with TRD and prescribed ESK-NS independent of the study. The study comprised a screening/baseline period, an on-treatment period (variable duration) and a 6- month post-treatment follow-up (**Fig 1**). Data were collected from routine clinical practice for clinical, social and economic outcomes, dosing, frequency and duration of treatment in clinical settings and pt outcomes. According to local regulations/clinical practice, pt-reported outcomes were also obtained.

Data collected at every visit included ESK-NS dosing/frequency, blood pressure before/after ESK-NS administration, concomitant medication/non-pharmacological treatments, time of and reasons for discontinuation (if applicable), and adverse events. At Weeks (Wks) 4, 8 and 12 (± 7 days), and then every 12 wks (± 15 days), data collection included various measures of depressive symptoms, clinical response, and pt-reported outcomes, including the Columbia Suicide Severity Rating Scale (C-SSRS). Montgomery-Åsberg Depression Rating Scale (MADRS) was assessed every 2 wks for 8 wks, then every 12 wks. Clinical management was reflective of local practice and at the sole discretion of the treating physician.

Statistical analyses will be descriptive (continuous variables) or summarised using frequencies and percentages (categorical variables).

**Results:**

571 pts were enrolled in the ECHO study (Germany: 145; Italy: 124; Israel: 101; Belgium: 89; Spain: 59; Sweden: 42; Netherlands: 11). Baseline characteristics were representative of a broad pt population with TRD (Constant *et al*. ECNP 2025 [PS02-1216]). Analysis is ongoing; preliminary study results regarding effectiveness and safety of ESK-NS in a real-world setting will be reported in the poster, in addition to comorbidities and post- treatment follow-up outcomes.

**Image 1:**
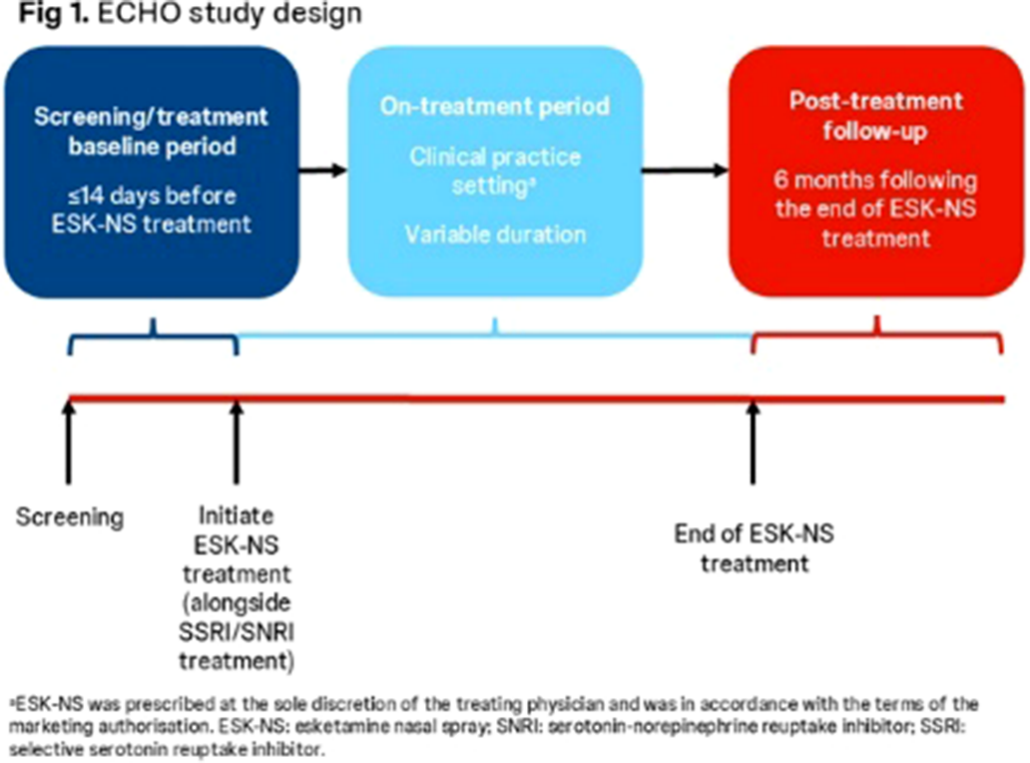
Image 1: Long description.

**Conclusions:**

ECHO is the first large prospective real-world evidence study of ESK-NS in TRD, with a broader pt population than previously studied. Data will enhance understanding of real- world ESK-NS use, which may improve guidance and treatment strategies for pts with TRD.

**Acknowledgements**. We thank the pts who participated. Funding: J&J; medical writing: Costello Medical.

**Disclosure of Interest:**

G. Rosso Consultant of: Angelini, Johnson & Johnson, Lundbeck, Neuraxpharm, Otsuka and Rovi., Speakers bureau of: Angelini, Johnson & Johnson, Lundbeck, Neuraxpharm, Otsuka and Rovi., E. Constant Grant / Research support from: AstraZeneca, BMS, Eli Lilly and Company, Janssen-Cilag, Lundbeck, Pfizer, Servier, Teva, Viatris and Wyeth., Consultant of: AstraZeneca, BMS, Eli Lilly and Company, Janssen-Cilag, Lundbeck, Pfizer, Servier, Teva, Viatris and Wyeth., Speakers bureau of: AstraZeneca, BMS, Eli Lilly and Company, Janssen-Cilag, Lundbeck, Pfizer, Servier, Teva, Viatris and Wyeth., S. De Filippis Grant / Research support from: Angelini, Lundbeck, Johnson & Johnson, Otsuka, Viatris, Molteni and Rovi, Consultant of: Angelini, Lundbeck, Johnson & Johnson, Otsuka, Viatris, Molteni and Rovi, Speakers bureau of: Angelini, Lundbeck, Johnson & Johnson, Otsuka, Viatris, Molteni and Rovi, Y. Godinov Shareholder of: Johnson & Johnson, Employee of: Johnson & Johnson, L. Häggström Grant / Research support from: Participated in clinical studies on esketamine, Consultant of: Johnson & Johnson and Lundbeck, Speakers bureau of: Johnson & Johnson and Lundbeck, A. Ibañez Grant / Research support from: Alter, Casen Recordati, Janssen-Cilag, Lundbeck, Otsuka Pharmaceutical SA, Rovi and Viatris, Consultant of: Alter, Casen Recordati, Janssen-Cilag, Lundbeck, Otsuka Pharmaceutical SA, Rovi and Viatris, Speakers bureau of: Alter, Casen Recordati, Janssen-Cilag, Lundbeck, Otsuka Pharmaceutical SA, Rovi and Viatris, Y. Kambarov Shareholder of: Johnson & Johnson, Employee of: Johnson & Johnson, E. Olivier Shareholder of: Author declared no conflicts of interest., J. M. Olivares Grant / Research support from: AstraZeneca, BMS, Eli Lilly and Company, Glaxo-Smith-Kline, Johnson & Johnson, Lundbeck, Novartis, Otsuka, Pfizer and Sanofi-Aventis, Consultant of: AstraZeneca, BMS, Eli Lilly and Company, Glaxo-Smith-Kline, Johnson & Johnson, Lundbeck, Novartis, Otsuka, Pfizer and Sanofi-Aventis, Speakers bureau of: AstraZeneca, BMS, Eli Lilly and Company, Glaxo-Smith-Kline, Johnson & Johnson, Lundbeck, Novartis, Otsuka, Pfizer and Sanofi-Aventis, C. Reif-Leonhard Consultant of: CAMPUS, Johnson & Johnson, LivaNova and Medice, Speakers bureau of: CAMPUS, Johnson & Johnson, LivaNova and Medice, B. Rive Shareholder of: Johnson & Johnson, Employee of: Johnson & Johnson, I. Turkoz Shareholder of: Johnson & Johnson, Employee of: Johnson & Johnson, C. von Holt Shareholder of: Johnson & Johnson, Employee of: Johnson & Johnson, D. Zeeuws Grant / Research support from: Participated in clinical trials on esketamine, Consultant of: Johnson & Johnson and Lundbeck, Speakers bureau of: Johnson & Johnson and Lundbeck, S. Köhler Grant / Research support from: Participated in clinical studies on esketamine, Consultant of: ARISTO Pharma, Johnson & Johnson, Medice and Otsuka, Speakers bureau of: ARISTO Pharma, Johnson & Johnson, Medice and Otsuka.

## Long descriptions

### Image 1: Long description

The flowchart consists of three main colored boxes at the top, connected by horizontal arrows from left to right.

1. The first box on the left is dark blue and labeled Screening/treatment baseline period, less than or equal to 14 days before E S K-N S treatment.

2. The middle box is light blue and labeled On-treatment period, Clinical practice setting, Variable duration.

3. The third box on the right is red and labeled Post-treatment follow-up, 6 months following the end of E S K-N S treatment.

Below these boxes is a horizontal red timeline with three vertical black arrows pointing upward to specific milestones:

- The first arrow on the far left is labeled Screening.

- The second arrow, aligned with the transition between the dark blue and light blue boxes, is labeled Initiate E S K-N S treatment (alongside S S R I/S N R I treatment).

- The third arrow, aligned with the transition between the light blue and red boxes, is labeled End of E S K-N S treatment.

Brackets above the timeline correspond to the three phases. A footnote at the bottom defines E S K-N S as esketamine nasal spray, S N R I as serotonin-norepinephrine reuptake inhibitor, and S S R I as selective serotonin reuptake inhibitor.

Navigate back to Image 1:.

